# Paracrine signalling of AGR2 stimulates RhoA function in fibroblasts and modulates cell elongation and migration

**DOI:** 10.1080/19336918.2019.1685928

**Published:** 2019-11-11

**Authors:** Hitesh Bhagavanbhai Mangukiya, Hema Negi, Siva Bharath Merugu, Qudsia Sehar, Dhahiri Saidi Mashausi, Fakhar-Un-Nisa Yunus, Zhenghua Wu, Dawei Li

**Affiliations:** a School of Pharmacy, Shanghai Jiao Tong University, Shanghai, China; b Engineering Research center of Cell and Therapeutic Antibody of Ministry of Education, Shanghai Jiao Tong University, Shanghai, China

**Keywords:** Anterior gradient 2, RhoA GTP-Binding Protein, fibroblasts, cell movement, Tumour microenvironment

## Abstract

The most prominent cancer-associated fibroblasts (CAFs) in tumor stroma is known to form a protective structure to support tumor growth. Anterior gradient-2 (AGR2), a tumor secretory protein is believed to play a pivotal role during tumor microenvironment (TME) development. Here, we report that extracellular AGR2 enhances fibroblasts elongation and migration significantly. The early stimulation of RhoA showed the association of AGR2 by upregulation of G1-S phase-regulatory protein cyclin D1 and FAK phosphorylation through fibroblasts growth factor receptor (FGFR) and vascular endothelial growth factor receptor (VEGFR). Our finding indicates that secretory AGR2 alters fibroblasts elongation, migration, and organization suggesting the secretory AGR2 as a potential molecular target that might be responsible to alter fibroblasts infiltration to support tumor growth.

## Introduction

Tumour microenvironment (TME) consists of complex interactions of tumour cells with the extracellular matrix (ECM). These phenomena are regulated by the exchange of biophysical and biochemical factors which contribute to detachment, migration, proliferation, invasion, and re-attachment of the cells present in TME [1]. The detailed analysis of these mechanisms is mandatory in the context of understanding mechanical processes such as cell adhesion, cell movements and motility, and the generation of forces within TME. Specifically, conventional two-dimensional (2D) and 3D *in vitro* models reveal that cancer-associated fibroblasts (CAFs) are involved in developing protective effect in order to contribute stroma formation and progression of TME development [1–4]. The stromal alterations are caused by an increased accumulation of fibroblasts and collagenized ECM. Recently published research work shows that various molecules and factors expressed by cancer cells regulate the accumulation and transformation of normal fibroblasts (NFs) to cancer-associated fibroblasts (CAFs) which develop as most prominent stromal cell type [5–7]. Cancer cells secrete various molecules like transforming growth factor-β (TGF-β), vascular endothelial growth factor (VEGF), basic fibroblast growth factor (bFGF), insulin-like growth factor-1 (IGF-1) and interleukin-6 [8–12]. These tumour niche secretome plays a pivotal role in cellular communications and thus regulates stromal fibroblasts to support tumour growth [13].

Anterior gradient 2 is a Xenopus XAG2 homolog protein [14,15], overexpressed and secreted into ECM by cancer cells has a pivotal role in TME formation [16]. AGR2 promotes cell migration, proposed as a potential drug target [17,18], and biomarker for circulating tumour cell detection [19,20]. Tumorigenic functions of AGR2 have been thoroughly investigated by many researchers [21]. Previously, we have reported the mechanism of extracellular AGR2 as a regenerative medicine which promotes cutaneous wound healing by recruitment of fibroblasts in the wounded area [19,21]. This finding suggests that AGR2 might be responsible for promoting fibroblasts recruitment and organization in TME. The tumour-related function of intracellular and secretory AGR2 has been investigated intensively in promoting angiogenesis and fibroblasts modulation in TME formation [22–25]. In tumorigenesis, AGR2 plays an important role by interacting with cyclin D1, cathepsin B, D, Myc, p-Src, and EGFR [26–28]. Very few functions of extracellular AGR2 have been reported explaining the fibroblasts coordinated tumour cell invasion and promotion of angiogenesis [16]. However, the extracellular AGR2 signalling mechanism underlying fibroblasts transformation, possible relation with cell cycle proteins and regulation in TME is still poorly understood. Moreover, how extracellular AGR2 passes its signal to upregulate and downregulate other cellular functional molecules like RhoA, Rac1, and CDC42 are still unknown. Especially, secretory AGR2 signalling pathway to nearby cells e.g. fibroblasts in ECM and initiation of cell regulation, migration, and organization by cross-talk among signalling molecules remains unknown.

In the context of TME, it is necessary to better understand the underlying molecular mechanisms of tumour cell secretion and as such AGR2 has been identified as a key player in such functions [18]. Based on previous studies, we assert that AGR2 secreted by tumour cells create a gradient in TME believed to regulate stromal cells like fibroblasts. We aimed to study the functional mechanism of extracellular AGR2 especially on fibroblasts by developing an AGR2 concentration gradient under soft agar DMEM (saDMEM). Here, we report that fibroblasts sprout and start migrating upon receiving signal by extracellular AGR2 gradient through FGFR and VEGFR. The temporal dynamic AGR2 concentration gradient showed enhancement of fibroblasts mobility and total migration. Our study demonstrates that AGR2 stimulates RhoA and CDC42 expression and has a possible relation with cell cycle protein cyclin D1 expression. We report that extracellular AGR2 execute its function by enhancing RhoA expression to phosphorylate FAK and cyclin D1 expression for fibroblasts proliferation, elongation, and migration. Our results indicate that secreted AGR2 is a potential anticancer therapeutic target to block the fibroblasts transformation and organization during the formation of ECM.

## Results

### Extracellular AGR2 increases the chemotaxis of NIH3T3 cells through FGFR and VEGFR under saDMEM

**Figure 1.**
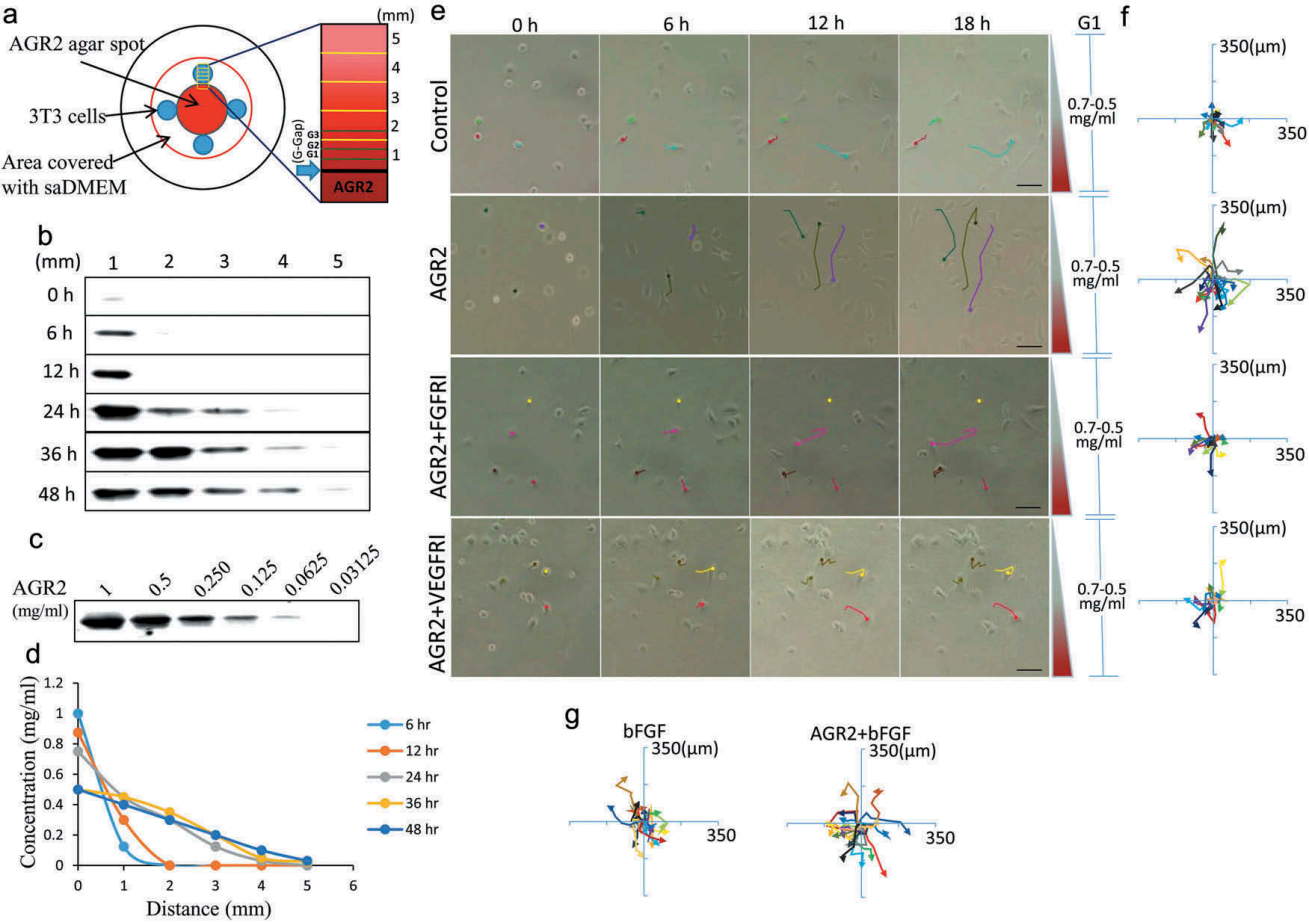
Extracellular AGR2 increases the chemotaxis of NIH3T3 cells through FGFR and VEGFR under saDMEM. (a) Schematic diagram showing generation and simulation of the AGR2 concentration gradient. (b) Western blot results showing AGR2 concentration gradient development compared with a western blot of (c) standard AGR2 concentration. (d) The plot of relative AGR2 concentration calculated from band intensity using Image studio software. (e) Time-lapsed images of NIH3T3 cells migrating along the concentration gradient of AGR2 in G1 under saDMEM semisolid medium, Images were taken using phase contrast microscopy at x100 magnification. Scale bar: 100 µm. (f) & (g) Trajectories of NIH3T3 cells migration along a concentration gradient of AGR2, AGR2-FGFRI, and AGR2-VEGFRI, bFGF, AGR2-bFGF. AGR2: anterior gradient 2; bFGF: basic fibroblast growth factor 2; FGFRI: fibroblast growth factor receptor inhibitor; VEGFRI: vascular endothelial growth factor receptor inhibitor.

A schematic diagram as shown in Figure 1(a) was designed to create AGR2 concentration gradient in saDMEM semisolid medium for individual cell migration analysis. Before conducting the experiment, we analysed the development of AGR2 concentration gradient by sampling the saDMEM at various time intervals from different distance points. The saDMEM samples were examined for the relative concentration of AGR2 by western blot analysis (Figure 1(b)) and comparing them with the band intensity of standard AGR2 (Figure 1(c)). According to the western blot results, the AGR2 protein was diffused from the centre (high concentration) to peripheral area (no concentration) forming a concentration gradient across the saDMEM semisolid medium starting from 0.125 mg/ml to 0.4 mg/ml (6 h to 48 h) linearly as shown in Figure 1(d). Thus, temporal dynamic AGR2 concentration gradient was developed from in saDMEM and we could investigate the mobility and migration of 3T3 cells under saDMEM extracellular matrix microenvironment.

We first examined the effect of AGR2, bFGF, and AGR2 coupled with bFGF on NIH3T3 cells chemotaxis by exposing cells to temporal dynamic concentration gradient in saDMEM semisolid medium. We found that NIH3T3 cells become mobile as soon as AGR2 gradient reaches the cells and start migrating towards the optimum concentration they require for the intracellular activity. The AGR2 concentration changes with time which impacts the migration of fibroblasts as shown in Figure 1(e). For cellular behaviour observation and quantification, the position of fibroblasts was tracked every 3h by overlaying time-lapsed images taken up to 18h for each condition. The trajectories of NIH3T3 cells along AGR2, bFGF, and AGR2 plus bFGF concentration gradient were plotted as shown in Figure 1(f–g). The preliminary trajectory data shows that NIH3T3 cells became more mobile along the AGR2 concentration gradient in saDMEM. However, when cells were exposed to a gradient of AGR2 coupled with bFGF, a significant increase in cell movement was observed. Furthermore, To investigate the first most signalling of AGR2, NIH3T3 cells were exposed to FGFR1 inhibitor PD173074 (FGFRI) and VEGFR inhibitor axitinib (VEGFRI) under saDMEM. The trajectory data shows that the mobility of NIH3T3 cells reduced significantly during inhibitor treatment and exhibits a reversed effect of AGR2 concentration gradient on chemotaxis. This result suggested that extracellular AGR2 increases the chemotaxis of fibroblasts through FGFR and VEGFR.

### Extracellular AGR2 coupled with bFGF increases the migration of 3T3 cells

**Figure 2.**
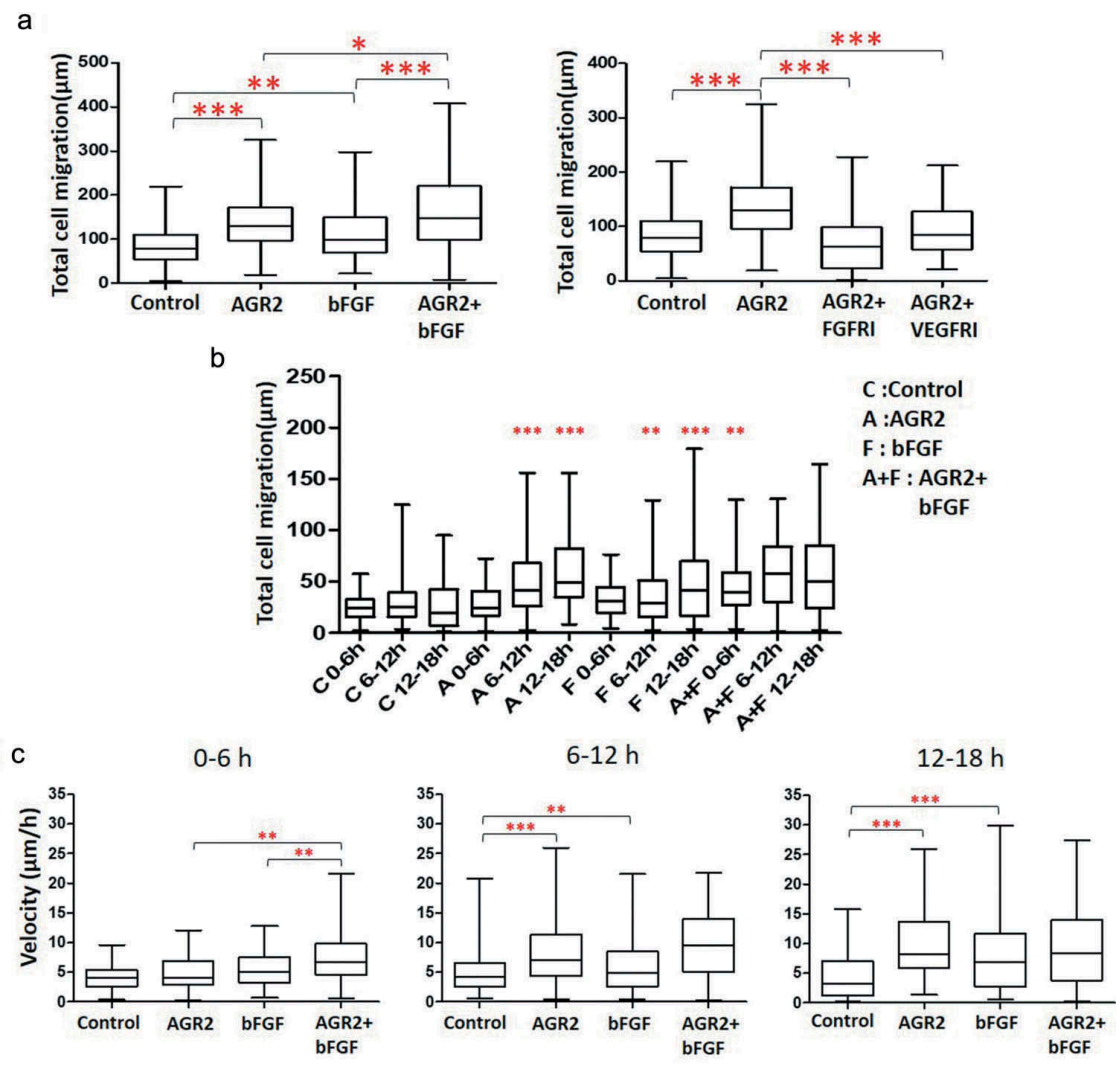
Extracellular AGR2 coupled with bFGF increases the migration of 3T3 cells. (a) Migration of NIH3T3 cells along the concentration gradient after 18 h calculated from the trajectory data (n = 80). (b) Total Migration of NIH3T3 cells tracked at 6 h interval and analysed the mobility of cells (n = 80). (c) Migration speed of NIH3T3 cells calculated by tracking an individual cell from 0–6 h, 6–12 h, and 12–18 h (n = 80). Asterisks indicate a significant difference in cell migration. (*p < 0.01, **p < 0.001, ***p < 0.0001).

The trajectory data was used for statistical analysis to calculate the total migration of the NIH3T3 cells. According to the quantitative analysis, the total distance travelled by NIH3T3 cells increased 1.66 fold and 1.38 fold under the AGR2 and bFGF concentration gradient respectively. Additionally, we observed that the migration of NIH3T3 cells was increased 1.27 fold more in AGR2-bFGF concentration gradient compared to AGR2 and bFGF alone as shown in Figure 2(a). Additionally, statistical analysis of NIH3T3 cells treated with FGFRI and VEGFRI showed significantly reduced the effect of the AGR2 concentration gradient. The AGR2 concentration gradient developed in saDMEM was temporary which exerted an altered effect on individual NIH3T3 cells. Furthermore, we fractionated total migration of individual NIH3T3 fibroblasts to investigate the migration speed under saDMEM. We found that the migration of fibroblasts increased noticeably 1.47 fold with AGR2 coupled with bFGF during 0–6 h compared to the treatment of AGR2 and bFGF separately (Figure 2(b)). However, the migration speed and total distance travelled by the cells was almost equivalent in AGR2, bFGF and AGR2-bFGF treatment during 6–12 h as well as 12–18 h and significantly higher as compared to control (Figure 2(b–c)). This result suggests that AGR2 promotes the activity of bFGF and enhances the migration of fibroblasts.

### Effect of AGR2 on 3T3 cell migration is concentration dependent

**Figure 3.**
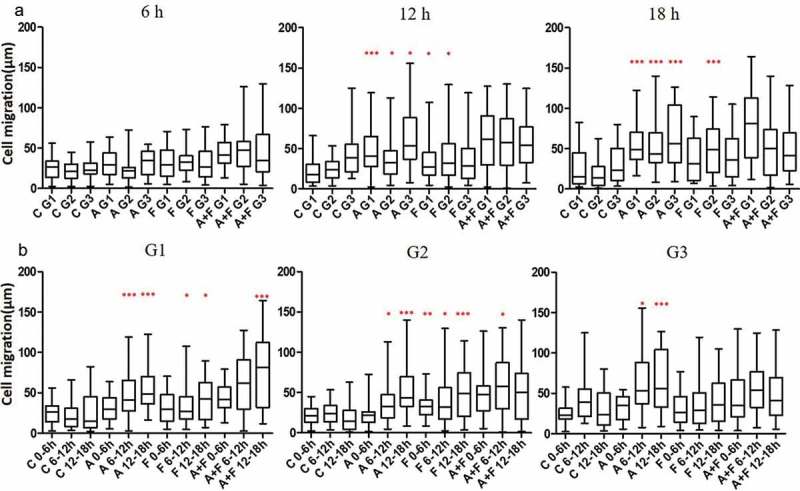
Effect of AGR2 on NIH3T3 cell migration is concentration dependent. (a) Individual cells migration after 6 h, 12h and 18 h in G1, G2, and G3 are plotted for each condition tested (n = 25). (b) The chemotaxis for average cells in G1, G2 and G3 after 6 h, 12h, and 18 h are plotted for each condition tested (n = 25). Asterisks indicate a significant difference in cell migration. (*p < 0.01, **p < 0.001, ***p < 0.0001). C: Control; A: AGR2; F: bFGF; A + F: AGR2-bFGF.

Extracellular AGR2 secreted by cancer cells creates a concentration gradient for the nearby cells in the tumour microenvironment. Cellular response to AGR2 stimulation occurs across various time scales. To demonstrate the gradient effect of AGR2 in NIH3T3 cells migration, we analysed cells present in Gap1 (G1), G2 and G3 after 6 h, 12 h and 18 h under saDMEM cell migration assay. As shown in Figure 3(a), there was no difference in the migration of cells up to 6 h compared to control. Interestingly, as soon as the AGR2 concentration gradient reached to the cells present in G1, G2, and G3, they travelled 2.15, 1.52, and 1.46 fold more compared to the cells in control during 6 h to 12 h, respectively. Additionally, cell migration became more significant as the AGR2 gradient developed to G3 became optimum during 12 h to 18 h. However, under bFGF concentration gradient cells present in G1 and G2 migrated obviously more during 6 h to 12 h and in G3 during 12 h to 18 h compare to control. There was no significant difference in migration of NIH3T3 cells under the concentration gradient created by AGR2, bFGF separately and AGR2-bFGF gradient.

When we analysed cells present in G1, G2 and G3 phase separately as shown in Figure 3(b), we found that 3T3 cells present in Gap 1 under AGR2-bFGF gradient migrated 2.13, 1.33, and 1.84 fold more as compared to control, AGR2, and bFGF group, respectively. In addition, cells present in Gap 2 under AGR2-bFGF gradient showed 2.42, 1.58, and 1.47 fold increased migration compare to control, AGR2, and bFGF gradient during 6 h to 12 h, respectively. Altogether, these results suggest that when 3T3 cells under saDMEM received an optimum concentration of AGR2 and bFGF through concentration gradient required to stimulate the NIH3T3 cells, the total cell migration was increased.

### Secretory AGR2 helps to organize fibroblasts by promoting cell elongation and proliferation

**Figure 4.**
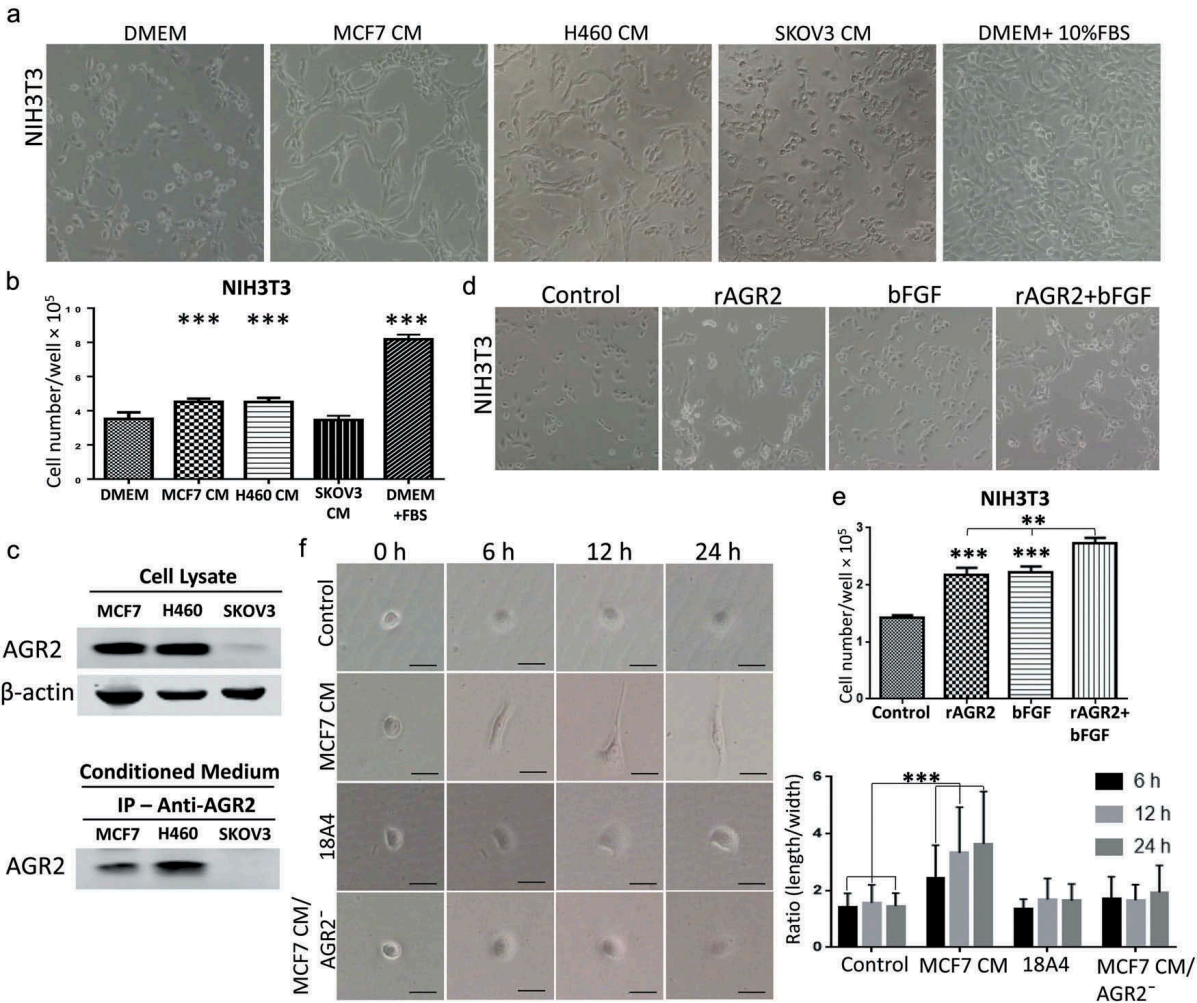
Secretory AGR2 helps to organize fibroblasts by promoting cell elongation and proliferation. (a) Effect of CM collected after 5 days from MCF7, H460, SKOV3 cultures on the growth of 3T3 cells, observed after 24 h using phase contrast microscopy at x100 magnification. (b) The proliferation of NIH3T3 cells plotted after 24 h by counting cells using haemocytometer. (c) The expression of AGR2 analysed from MCF7, H460 and SKOV3 cell lysate and conditioned medium by western blot and immunoprecipitation as indicated. 50 ml conditioned medium of each culture was used for immunoprecipitation. (d) Effect of rAGR2, bFGF, and rAGR2-bFGF on the growth of 3T3 observed using phase contrast microscopy at x100 magnification. The concentration of rAGR2 is 500 ng/ml. The concentration of bFGF is 1 ng/ml. (e) The proliferation of 3T3 plotted after 24 h by counting cells using haemocytometer. Each column represents mean±SD (n = 5). (f) Cells were seeded and treated with MCF7 CM, 18A4 mAb and MCF7 CM/AGR2¯ and typical morphology were obtained by phase contrast microscopy. Scale bar: 100 µm (g) Ratio (length/width) was obtained using Image-Pro Plus software by using 80 isolated cells for each condition. Asterisks indicate the significant difference of cells proliferation and elongation. (**p < 0.001, ***p < 0.0001).

Cancer-associated fibroblasts are a major constituent of tumour microenvironment and commonly display myofibroblastic characteristics, which play a vital role in promoting cancer progression. To demonstrate the effect of AGR2 secreted by cancer cells on fibroblasts organization and for morphological observation, we treated NIH3T3 cells with conditioned medium (CM) collected after 5 days of MCF7, H460 and SKOV3 cultures for 24 h separately. We observed that fibroblasts were organized linearly and formed a complex matrix structure when treated with MCF7 CM and H460 CM. However, there was not any morphological change observed in SKOV3 CM treatment (Figure 4(a)). Interestingly, fibroblasts elongation was reduced significantly when secretory AGR2 was pulled out from CM by immunoprecipitation using 18A4 mAb (Anti-AGR2 antibody) (Supplementary Figure 1). To see the proliferative effect of MCF7 CM, H460 CM, and SKOV3 CM, we harvested cells and counted by haemocytometer. We found that the proliferation of NIH3T3 cells was increased significantly as shown in Figure 4(b). To correlate this effect whether it was actually caused by secretory AGR2, we immunoprecipitated the secretory AGR2 from conditioned medium using anti-AGR2 monoclonal antibody and checked through western blot. To our expectation, we observed AGR2 expression and secretion only in MCF7 and H460 cells, whereas there was no expression of AGR2 in SKOV3 cells as shown in Figure 4(c). To confirm our theory, we treated NIH3T3 cells with rAGR2, bFGF, and rAGR2-bFGF. We found linearization of NIH3T3 cells in rAGR2 alone and rAGR2 coupled with bFGF treatment (Figure 4(d)). Additionally, we observed significantly increased proliferation of fibroblasts in rAGR2-bFGF (1.97 fold) treatment compare to rAGR2 (1.53 fold) and bFGF (1.56 fold) alone (Figure 4(e)). These observations indicate that AGR2 interact with fibroblasts to organize in TME.

To understand the effect of secretory AGR2 on an individual cell, NIH3T3 cells treated with MCF7 CM, 18A4 mAb and MCF7 CM/AGR2¯ from which AGR2 was pulled out by immunoprecipitation using 18A4 mAb. After 6 h, cells treated with MCF7 CM were begun to exhibit noticeable thin projection and elongation. Many distinct filopodiae and protrusions consistent with migratory activity were observed after 12 h and 24 h in MCF7 CM treatment. On the opposite, NIH3T3 cells treated with MCF7 CM/AGR2¯ displayed round spread morphology and failed to extend filopodiae and elongation (Figure 4(f)). To support this observation, the number of elongated cells was quantified. As shown in Figure 4(g), quantitative analysis by measurement of the length-width ratio of NIH3T3 cells treated with MCF7 CM showed obvious cell elongation compare to control. Additionally, a significant reduction of cell elongation in MCF7 CM/AGR2¯ treatment was also observed. Altogether, our results suggest that, as soon as NIH3T3 fibroblast receives signal of AGR2, cells get activated and begin to elongate for migration and proliferation for the linear cellular organization which is believed to promote tumour growth.

### Extracellular AGR2 enhances RhoA, CDC42 expression and stimulates FAK phosphorylation through FGFR and VEGFR in NIH3T3 cells

**Figure 5.**
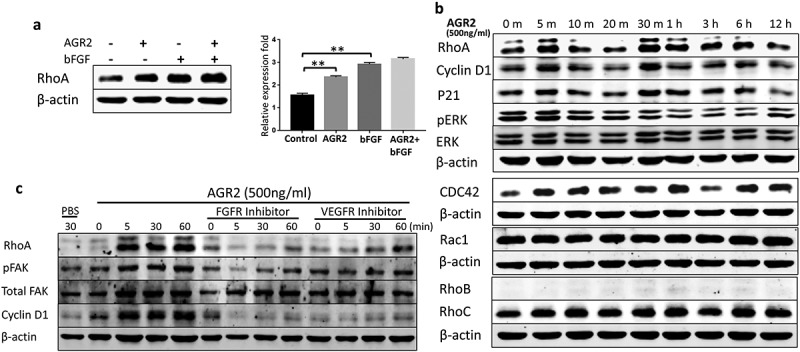
Extracellular AGR2 enhances RhoA, CDC42 expression and stimulates FAK phosphorylation in NIH3T3 cells. (a) Western blot analysis of RhoA expression in NIH3T3 cells stimulated for 24 h with AGR2 coupled with or without bFGF. The concentration of AGR2 is 500 ng/ml. The concentration of bFGF is 1 ng/ml. (b) NIH3T3 cells were treated by 500 ng/ml AGR2 for the indicated time. RhoA, cyclin D1, P21, ERK1/2, pERK1/2, CDC42, Rac1, RhoB, and RhoC were detected by western blots. β-actin served as loading control. (c) Significant reduction in RhoA expression, cyclin D1 expression, and FAK phosphorylation was observed in the NIH3T3 cells pretreated with 20 nM/ml FGFR1 inhibitor PD173074 and 10 nM/ml VEGFR inhibitor axitinib before AGR2 treatment. β-actin served as loading control.

**Figure 6.**
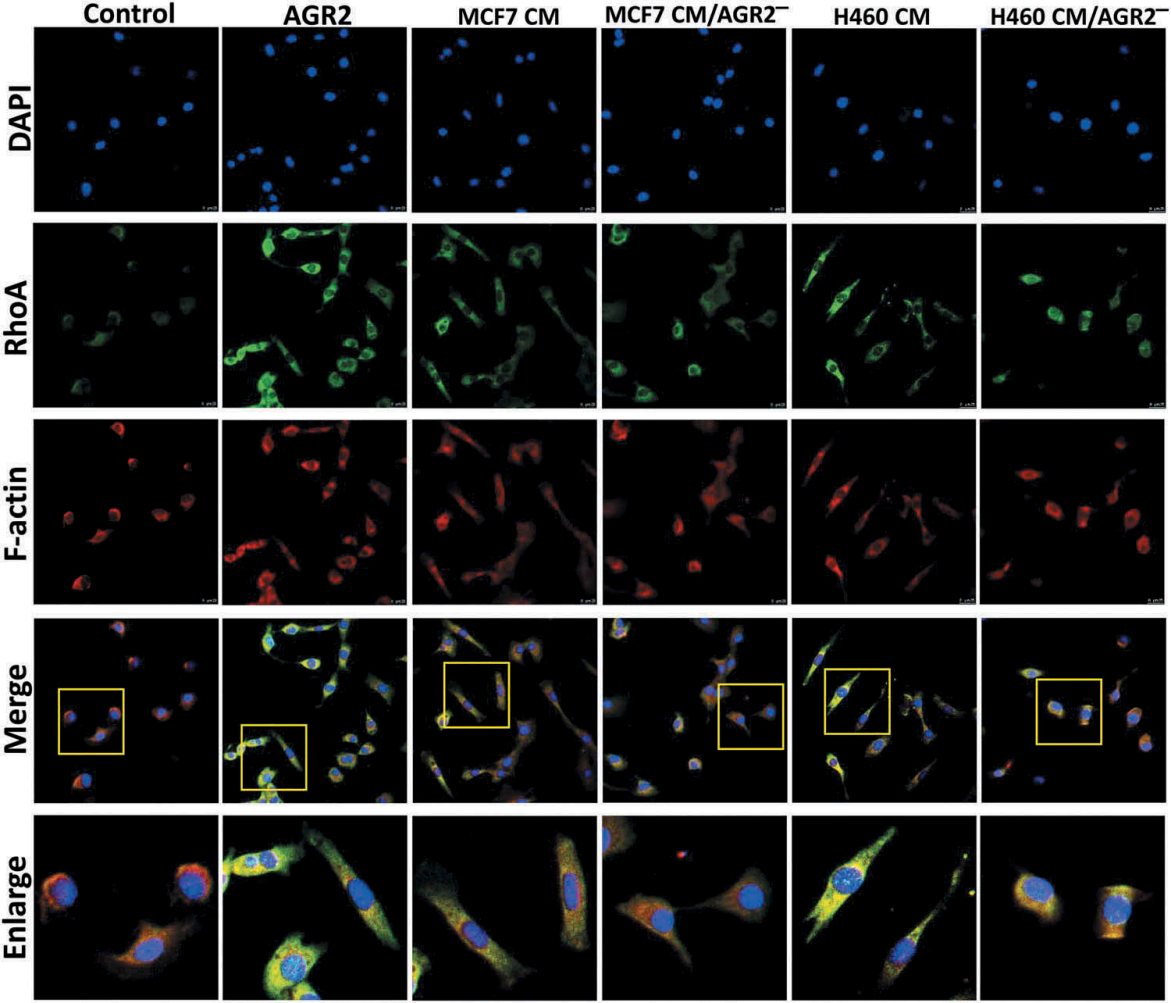
Secretory AGR2 enhances RhoA expression and induces NIH3T3 cells elongation. (a) NIH3T3 cells were treated with AGR2 (500ng/ml), MCF7CM, MCF7 CM/AGR2̅, H460 CM and H460 CM/AGR2 ® for 24 h and immunostained for RhoA and F-actin. Scale bar: 25 µm. Bottom row showing enlarge area indicated in merge image panel.

Recently, AGR2 has been investigated for interaction with cell cycle protein cyclin D1 and p21 that alters p53 function in ovarian cancer [22,29,30]. However, the possible relation and molecular mechanism of AGR2 with cytoskeletal regulatory factors RhoA, CDC42, Rac1 and association with cell cycle protein cyclin D1 expression are unknown in the context of fibroblasts. NIH3T3 cells were treated with AGR2, bFGF, and AGR2-bFGF for 24h. The western blot result showed that AGR2 stimulated the activation of RhoA in NIH3T3 cells. Interestingly, we observed slightly elevated expression of RhoA when treated with AGR2 coupled with bFGF as compared to the individual treatment of AGR2 and bFGF (Figure 5(a)). In order to evaluate the RhoA upregulation, NIH3T3 cells were treated for the indicated time as shown in Figure 5(b). The western blot results demonstrated an early response to AGR2 by increased RhoA expression in just 5 minutes and again showing high expression at 30 min. Additionally, we detected high expression of CDC42 by AGR2 which was corresponding to RhoA expression. However, there was not any significant change in expression of Rac1, RhoB and RhoC observed by AGR2. The early stimulation of RhoA was correlated with G1-S phase regulatory protein cyclin D1 expression (Figure 5(b)). In cell migration, the formation of focal adhesions involves the coordinated action of various classes of molecules, in which FAK was reported to play a key role in assembly and disassembly of the focal adhesions. The FAK is the downstream signalling molecule of RhoA signalling cascade, therefore we examined the correlation of hypothetical AGR2-RhoA-FAK signalling cascade to investigate the whole signalling pathway of extracellular AGR2 for its function on cell proliferation and migration. To confirm this, NIH3T3 cells were pretreated with FGFRI and VEGFRI and we found significantly FAK phosphorylation which is correlated with the reduced RhoA expression, cyclin D1 expression (Figure 5(c)). This result indicates that AGR2 might be altering the morphology and mobility of NIH3T3 cells by stimulating RhoA and upregulating cyclin D1 expression and FAK phosphorylation. Further evaluation by immunofluorescence staining demonstrated that secretory AGR2 stimulates RhoA expression significantly which is correlated with the cell elongation and migratory cells as shown in Figure 6.

**Figure 7.**
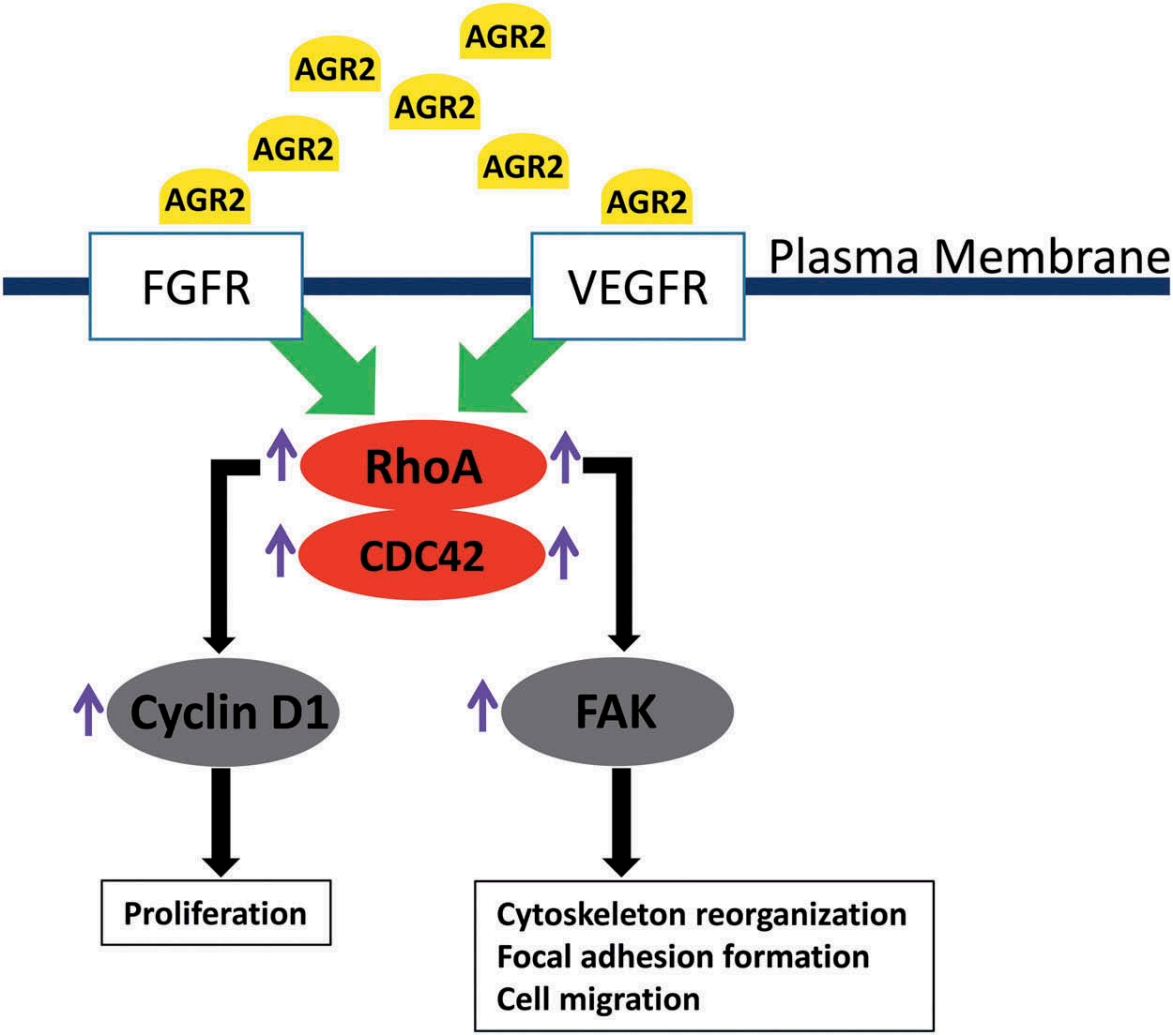
Hypothetic model of paracrine signalling of AGR2 in fibroblasts. In the initial signal, extracellular AGR2 could stimulate RhoA expression through FGFR and VEGFR. As a signalling cascade, increased RhoA expression upregulated G1-S phase cell cycle molecule cyclin D1 for proliferation mechanism. Another mechanism of AGR2 through RhoA expression could phosphorylate FAK for cytoskeleton reorganization and focal adhesion formation to promote cell migration.

Thus, extracellular AGR2 passes the signal through FGFR and VEGFR to enhance the function of RhoA, which upregulates cyclin D1 and FAK phosphorylation. This signalling pathway results lead us to hypothesize an unknown molecular mechanism of tumour niche secretome AGR2 as shown in Figure 7, which might be responsible to recruit/activate non-transformed host cells like fibroblasts for tumour niche microenvironment progression.

## Discussion

As per the biochemical and biophysical phenomenon, the secretory factors from the tumour cells initiate the cross talk among ECM and tumour cells. It has been reported by many researchers that fibroblasts are important constituents of tumour stroma [4,31–33]. NIH3T3 has been used to study tumour progression in heterotypic tumour models [2,34–37]. Based on this research, we explored the gradient effect of extracellular AGR2 on NIH3T3 fibroblasts. Our results demonstrated that extracellular AGR2 enhanced chemotaxis of NIH3T3 fibroblasts along the AGR2 concentration gradient under saDMEM semisolid medium. The significantly reversed effect of AGR2 on fibroblasts migration indicated that AGR2 paracrinely passes its signal through FGFR and VEGFR. Our previous study showed that AGR2 acts like a chaperone and binds with bFGF for tumorigenic activity [16]. In this study, we demonstrated an individual cell analysis, which also showed AGR2 enhanced bFGF activity and increased NIH3T3 fibroblasts migration significantly.

Functional AGR2 is effective only around the AGR2-secreting cells where tumour-secreted proteins in tumour interstitial fluid have different concentration magnitudes [38,39]. We established temporal dynamic AGR2 concentration gradient using saDMEM to examine the gradient effect of AGR2 exposed to fibroblasts. In our investigation, we reported that NIH3T3 fibroblasts became highly mobile and started migration when it received an optimum concentration of AGR2 required for the migratory activity. However, the mobility of fibroblasts which could not receive optimum AGR2 signal was the same as in control cells. Cell migration is controlled by the physical and extracellular modulators such as ECM alignment, stiffness by components, flexibility, density and crosstalk among ECM components [40–42]. In our investigation, an individual cell migration analysis showed that only cells which received optimum threshold concentration of AGR2 were activated and showed significant migration. This result is supported by blocking FGFR and VEGFR demonstrating reduced migration of NIH3T3 cells. This shows that each fibroblast behaves differently when extracellular AGR2 exerts its functional effect through FGFR and VEGFR, which might be related to the morphological transformation of fibroblasts.

Stromal fibroblasts have been reported to create a physical barrier by the formation of a compact matrix structure to develop tumour spheroid [43–47]. In our study, we reported that MCF7 and H460 cancer cell secreted AGR2 linearized NIH3T3 fibroblasts led to the formation of complex structures, which did not occur when cells were treated with SKOV3 CM. SKOV3 cells are bFGF positive cells and our results show that bFGF has no morphological effect on fibroblasts. However, AGR2 coupled with bFGF showed a more complex organization of the fibroblasts. Our investigation alludes that secretory AGR2 alters the fibroblasts morphology and organizes to form a complex structure.

Prior studies showed that small guanosine triphosphatases such as RhoA affect morphology and chemotactic behaviour of cells [48–53]. Previously, we have demonstrated that AGR2 promotes cell invasion through JNK-MAPK pathway to regulate focal adhesion assembly and disassembly [19]. In our current study, we report for the first time that extracellular AGR2 stimulates the RhoA and CDC42 expression in NIH3T3 fibroblasts. Furthermore, our results show that AGR2 coupled with bFGF enhance the expression of RhoA slightly more than AGR2 and bFGF separate treatment. Moreover, western blot results demonstrate that AGR2 could activate proliferative mechanisms such as increased expression of cyclin D1 and leading to its possible association with RhoA expression. Focal adhesion kinase has been studies as a scaffold RhoA activable signalling scaffold mediating Akt (Protein kinase B) activation and cardiomyocyte protection [54]. The correlation of RhoA expression and FAK phosphorylation by AGR2 through FGFR and VEGFR demonstrates the mechanism of cell migration. Thus, hypothetically AGR2 might be passing its signal through FGFR and VEGFR paracrinely to increase RhoA expression followed by upregulation of cyclin D1 and FAK phosphorylation (Figure 7). This result suggests that AGR2 influences RhoA expression, cyclin D1 expression, and FAK phosphorylation which in turn might be regulating NIH3T3 proliferation, cytoskeleton organization, and cell movements.

Morphology of NIH3T3 cells cultured in MCF7 CM appears different from the cells in DMEM supplemented with 10% serum. NIH3T3 cells on plastic surface displayed spindle-shaped or stellate phenotype under saDMEM semisolid medium and elongated with thinner and longer protrusions like dendritic extensions at the migration front when treated with MCF7 CM. Interestingly, our results show that NIH3T3 cells failed to produce cell elongation when AGR2 was pulled out from MCF7 CM and H460 CM by immunoprecipitation with AGR2 specific monoclonal antibody 18A4. Altogether, our investigation emphasizes the role of extracellular AGR2 on NIH3T3 fibroblasts regulation in ECM stroma and might be responsible for the mesenchymal phenotype acquisition by fibroblasts. Further understanding of this phenomenon might lead to AGR2 targeted antibody-based anti-tumour therapy.

## Materials and methods

### Cell culture

Mouse embryonic fibroblasts NIH/3T3, human breast adenocarcinoma MCF7, lung adenocarcinoma H460 and ovarian carcinoma SKOV3 cells were cultured and maintained in DMEM with 10% FBS, 1% penicillin and streptomycin (Solarbio, Beijing, China) inside a humidified 5% CO2 incubator at 37°C. All the experiments were performed in serum-free DMEM with 1% PS for AGR2 and bFGF treatment. For individual cell migration analysis saDMEM semisolid medium was formulated to create a concentration gradient of growth factors.

### Formulation of soft agar DMEM semisolid medium and experiment design

The soft agar DMEM (saDMEM) semisolid medium was formulated by mixing 2 ml of 1.8% agar with 5 ml of 2X DMEM making up the final volume up to 10ml with sterile distilled water. DMEM, 1.8% agar, and saDMEM were kept at 45°C in a water bath. The final concentration of the agar in soft agar DMEM semisolid was 0.35%. To create the concentration gradient, 100µl of soft agar DMEM semisolid medium containing 1 mg/ml AGR2 was released dropwise at the centre of well in a 6 well plate. After solidification, the well was filled with 1 ml of soft agar DMEM. The samples were taken from 1, 2, 3, 4, 5 mm distance (Figure 1(a)) at 6 h, 12 h, 24 h, 36 h, and 48 h. Samples were analysed by performing western blot using rAGR2 as a standard to evaluate the formation of a concentration gradient.

### Cell migration under saDMEM and cell tracking analysis

The saDMEM was prepared as described above and kept at 45°C in a water bath. To create the concentration gradient, a high concentration of AGR2 (1mg/ml), bFGF (1ug/ml) and AGR2-bFGF were mixed with saDMEM and 100µl of the mixture was dropped at the centre of an individual well in 6 well plates. The concoction was allowed to solidify. NIH3T3 fibroblasts at (3 × 10^5^ cells/ml) were suspended in DMEM and dropped 20 µl around the centre saDMEM drop containing growth factors. For FGFR inhibitor treatment 20ng/ml PD173074 and VEGFR inhibitor treatment 2ng/ml axitinib were mixed with the cell suspension. After 4h of incubation, the medium above the cells was removed, washed with 20µl serum-free DMEM and covered with 20µl saDMEM. After covering the cells, the whole area in the well was covered with 1 ml of saDMEM.

The time-lapse images of the cell movements were taken in phase contrast (10X magnification) at an interval of 3 h continuing up to 18 h. The time-lapse images were overlapped using Photoshop software to obtain the trajectories of fibroblasts migration under saDMEM. The images were partitioned into Gap1, Gap2 and Gap3 region of 250µm each to analyse the cells present in that specific area. The cell trajectories were used to calculate the distance travelled by fibroblasts along the concentration gradient of growth factors using Image-pro plus software and ImageJ software.

### Immunoprecipitation

Immunoprecipitation of AGR2 from MCF7 CM, H460 CM, and SKOV3 CM was performed as described previously with minor modifications [16]. To check the secretory AGR2, 60ml of CM obtained after 5 days from each of the cell culture was incubated with 15µg of 18A4 mAb and incubated for 4 h at 4°C on shaker. Then, 20µl of protein G beads (Thermo Scientific, Massachusetts, USA) were added and incubated for overnight at 4°C in shaking condition. After incubation, the mixture was centrifuged at 10,000 rpm for 20 min. The beads were washed three times with phosphate buffer and dissolved in sample loading buffer by boiling at 95°C hot water bath. For cell elongation experiment, MCF7 supernatant was filtered through a sterile 0.22 µm syringe filter (Millipore, Billerica, MA, USA).

### Cell morphology and proliferation assay

NIH3T3 cells were digested with trypsin and seeded at a density of 50,000 cells/well in 12 well plate or 20,000 cells per well in 24 well plate. Cells were starved for 24 hrs in serum-free medium. After starvation, in 12 wells plate, NIH3T3 cells were treated with conditioned medium (CM) obtained from MCF7, H460 and SKOV3 cells cultured for 5 days. After starvation, in 24 well plates, cells were treated with 500ng/ml AGR2, 1 ng/ml bFGF and AGR2-bFGF for 24 h. Experiments were performed in triplicate along with control in which cells were grown in serum- free medium as well as a respective standard medium.

### Cell elongation assay

NIH3T3 cells were seeded at a density of 10,000 cells/well in 12 well plate. After 6 hr, cells were treated with DMEM, MCF7CM, 18A4 mAb and MCF7 CM/AGR2¯ (AGR2 pulled out from MCF7 CM by immunoprecipitation) and time-lapse images were taken at 200X magnification by phase contrast microscopy at 0 h, 6 h, 12 h and 24 h of incubation. The ratio of length/width was calculated by image pro plus software as described previously [55].

### Western blot analysis

For RhoA expression studies, cells were starved in serum-free DMEM for 24 h and then exposed to 500 ng mL^−1^ AGR2 for indicated timings (Figure 5(b)) and 1 ng mL^−1^ bFGF, AGR2-bFGF for 24 h (Figure 5(a)). For inhibitor treatment cells were pre-treated with 20 nM/ml FGFR inhibitor PD173074 and 10 nM/ml VEGFR inhibitor axitinib. Whole cell lysates were prepared in lysis buffer containing 1%NP-40, 50 mM Tris-HCl (pH-8.0), and NaCl with 0.1% protease inhibitor (Merck, Kenilworth, NJ, USA) and 1% phosphatase inhibitor (Roche, Basel, Switzerland). Immunoblotting was performed as described previously with RhoA, p-FAK, Cyclin D1, p-ERK, ERK, P21 primary antibody (Cell signalling technology Inc., Denver, MA, USA), FAK, RhoB, RhoC, Rac1 Primary antibody (ABclonal, Woburn, MA, USA), CDC42 primary antibody (Abcam, Cambridge, MA, USA) and β-actin purchased from Abgent [16].

For AGR2 concentration gradient analysis samples of saDMEM and rAGR2 standards were resolved in each lane of 12% SDS-PAGE gel before transferring to the nitrocellulose membrane. Immunoblotting was performed with the anti-AGR2 antibody (Rabbit, Abgent; 1:1000). Protein bands were detected and analysed using Image studio Lite version 5.2 (LI-COR, Lincoln, NE, USA).

### Immunostaining

NIH3T3 cells were seeded on to the coverslips in 12 well plates and treated with AGR2, MCF7 CM, MCF7 CM/AGR2^−^, H460 CM and H460 CM/AGR2^−^ for 24 h. Cells were stained for RhoA and F-actin by incubating with Rabbit anti-RhoA primary antibody (Cell signalling technology, Inc. Denver, MA, USA) and Phalloidin (Millipore, Billerica, MA, USA) followed by Dylight goat anti-rabbit 488 antibody against RhoA primary antibody. Coverslips were mounted on the glass slide for confocal microscopic analysis.

### Statistical analysis

All of the experiments were performed in triplicates. The data was analysed statistically using one way ANOVA and Student’s *t*-test with GraphPad Prism (Graphpad Software Inc., La Jolla, CA, USA). *P* ˂ 0.01 was considered for statistical significance.

## References

[1] Ungefroren H, Sebens S, Seidl D, et al Interaction of tumor cells with the microenvironment. Cell Commun Signal. 2011 9;13(9):18. 10.1186/1478-811X-9-18 PMC3180438 21914164

[2] Lee SW, Kwak HS, Kang MH, et al Fibroblast-associated tumour microenvironment induces vascular structure-networked tumouroid. Sci Rep. 2018 2 5;8(1):2365. 29403007 10.1038/s41598-018-20886-0 PMC5799156

[3] Wong T, McGrath JA, Navsaria H. The role of fibroblasts in tissue engineering and regeneration. Br J Dermatol. 2007 6;156(6):1149–1155. 17535219 10.1111/j.1365-2133.2007.07914.x

[4] Kalluri R, Zeisberg M Fibroblasts in cancer. Nat Rev Cancer. 2006 5;6(5):392–401. 16572188 10.1038/nrc1877

[5] Sadlonova A, Novak Z, Johnson MR, et al Breast fibroblasts modulate epithelial cell proliferation in three-dimensional in vitro co-culture. Breast Cancer Res. 2005;7(1):R46–59. 15642169 10.1186/bcr949 PMC1064098

[6] Cohen N, Shani O, Raz Y, et al Fibroblasts drive an immunosuppressive and growth-promoting microenvironment in breast cancer via secretion of Chitinase 3-like 1. Oncogene. 2017 8;36(31):4457–4468. 28368410 10.1038/onc.2017.65 PMC5507301

[7] Hanahan D, Coussens LM Accessories to the crime: functions of cells recruited to the tumor microenvironment. Cancer Cell. 2012 3 20;21(3):309–322. 22439926 10.1016/j.ccr.2012.02.022

[8] Guo H, Chen H, Zhu Q, et al A humanized monoclonal antibody targeting secreted anterior gradient 2 effectively inhibits the xenograft tumor growth. Biochem Biophys Res Commun. 2016 6 17;475(1):57–63. 27166154 10.1016/j.bbrc.2016.05.033

[9] Hong XY, Wang J, Li Z AGR2 expression is regulated by HIF-1 and contributes to growth and angiogenesis of glioblastoma. Cell Biochem Biophys. 2013;67(3):1487–1495. 23712868 10.1007/s12013-013-9650-4

[10] Tsuji T, Satoyoshi R, Aiba N, et al Agr2 mediates paracrine effects on stromal fibroblasts that promote invasion by gastric signet-ring carcinoma cells. Cancer Res. 2015 1 15;75(2):356–366. 25488752 10.1158/0008-5472.CAN-14-1693

[11] Beacham DA, Cukierman E Stromagenesis: the changing face of fibroblastic microenvironments during tumor progression. Semin Cancer Biol. 2005 10;15(5):329–341. 15970443 10.1016/j.semcancer.2005.05.003

[12] Iacopino F, Angelucci C, Sica G Interactions between normal human fibroblasts and human prostate cancer cells in a co-culture system. Anticancer Res. 2012 5;32(5):1579–1588. 22593435

[13] Delom F, Nazaraliyev A, Fessart D The role of protein disulphide isomerase AGR2 in the tumour niche. Biol Cell. 2018 12;110(12):271–282. 30238476 10.1111/boc.201800024

[14] Aberger F, Weidinger G, Grunz H, et al Anterior specification of embryonic ectoderm: the role of the Xenopus cement gland-specific gene XAG-2. Mech Dev. 1998 3;72(1–2):115–130. 9533957 10.1016/s0925-4773(98)00021-5

[15] Kumar A, Godwin JW, Gates PB, et al Molecular basis for the nerve dependence of limb regeneration in an adult vertebrate. Science. 2007 11 2;318(5851):772–777. 17975060 10.1126/science.1147710 PMC2696928

[16] Guo H, Zhu Q, Yu X, et al Tumor-secreted anterior gradient-2 binds to VEGF and FGF2 and enhances their activities by promoting their homodimerization. Oncogene. 2017 9 7;36(36):5098–5109. 28481872 10.1038/onc.2017.132

[17] Salmans ML, Zhao F, Andersen B The estrogen-regulated anterior gradient 2 (AGR2) protein in breast cancer: a potential drug target and biomarker. Breast Cancer Res. 2013 4 24;15(2):204. 23635006 10.1186/bcr3408 PMC3672732

[18] Alkasalias T, Moyano-Galceran L, Arsenian-Henriksson M, et al Fibroblasts in the tumor microenvironment: shield or spear? Int J Mol Sci. 2018 5 21;19(5):1532. 10.3390/ijms19051532 PMC5983719 29883428

[19] Zhu Q, Mangukiya HB, Mashausi DS, et al Anterior gradient 2 is induced in cutaneous wound and promotes wound healing through its adhesion domain. Febs J. 2017 9;284(17):2856–2869. 28665039 10.1111/febs.14155

[20] Smirnov DA, Zweitzig DR, Foulk BW, et al Global gene expression profiling of circulating tumor cells. Cancer Res. 2005 6 15;65(12):4993–4997. 15958538 10.1158/0008-5472.CAN-04-4330

[21] Wang Z, Hao Y, Lowe AW The adenocarcinoma-associated antigen, AGR2, promotes tumor growth, cell migration, and cellular transformation. Cancer Res. 2008 1 15;68(2):492–497. 18199544 10.1158/0008-5472.CAN-07-2930

[22] Chevet E, Fessart D, Delom F, et al Emerging roles for the pro-oncogenic anterior gradient-2 in cancer development. Oncogene. 2013 5 16;32(20):2499–2509. 22945652 10.1038/onc.2012.346

[23] Brychtova V, Mohtar A, Vojtesek B, et al Mechanisms of anterior gradient-2 regulation and function in cancer. Semin Cancer Biol. 2015;33:16–24. 25937245 10.1016/j.semcancer.2015.04.005

[24] Higa A, Mulot A, Delom F, et al Role of pro-oncogenic protein disulfide isomerase (PDI) family member anterior gradient 2 (AGR2) in the control of endoplasmic reticulum homeostasis. J Biol Chem. 2011 12 30;286(52):44855–44868. 22025610 10.1074/jbc.M111.275529 PMC3248018

[25] Negi H, Merugu SB, Mangukiya HB, et al Anterior Gradient-2 monoclonal antibody inhibits lung cancer growth and metastasis by upregulating p53 pathway and without exerting any toxicological effects: a preclinical study. Cancer Lett. 2019 5 1;449:125–134. 30685412 10.1016/j.canlet.2019.01.025

[26] Kim SJ, Kim DH, Kang D, et al Expression of anterior gradient 2 is decreased with the progression of human biliary tract cancer. Tohoku J Exp Med. 2014 9;234(1):83–88. 25186196 10.1620/tjem.234.83

[27] Di Maro G, Salerno P, Unger K, et al Anterior gradient protein 2 promotes survival, migration and invasion of papillary thyroid carcinoma cells. Mol Cancer. 2014 6 30;13:160. 24976026 10.1186/1476-4598-13-160 PMC4094684

[28] Huang J, Wang L, Jiang M, et al AGR2-mediated lung adenocarcinoma metastasis novel mechanism network through repression with interferon coupling cytoskeleton to steroid metabolism-dependent humoral immune response. Cel Immunol. 2014 7;290(1):102–106. 10.1016/j.cellimm.2014.05.008 24960290

[29] Pohler E, Craig AL, Cotton J, et al The Barrett’s antigen anterior gradient-2 silences the p53 transcriptional response to DNA damage. Mol Cel Proteomics. 2004 6;3(6):534–547. 10.1074/mcp.M300089-MCP200 14967811

[30] Willis S, Villalobos VM, Gevaert O, et al Single gene prognostic biomarkers in ovarian cancer: a meta-analysis. PloS One. 2016;11(2):e0149183. 26886260 10.1371/journal.pone.0149183 PMC4757072

[31] Haviv I, Polyak K, Qiu W, et al Origin of carcinoma associated fibroblasts. Cell Cycle. 2009 2 15;8(4):589–595. 19182519 10.4161/cc.8.4.7669

[32] Mueller MM, Fusenig NE Friends or foes - bipolar effects of the tumour stroma in cancer. Nat Rev Cancer. 2004 11;4(11):839–849. 15516957 10.1038/nrc1477

[33] Ostman A, Augsten M Cancer-associated fibroblasts and tumor growth–bystanders turning into key players. Curr Opin Genet Dev. 2009 2;19(1):67–73. 19211240 10.1016/j.gde.2009.01.003

[34] Rama-Esendagli D, Esendagli G, Yilmaz G, et al Spheroid formation and invasion capacity are differentially influenced by co-cultures of fibroblast and macrophage cells in breast cancer. Mol Biol Rep. 2014 5;41(5):2885–2892. 24469725 10.1007/s11033-014-3144-3

[35] Saito RA, Micke P, Paulsson J, et al Forkhead box F1 regulates tumor-promoting properties of cancer-associated fibroblasts in lung cancer. Cancer Res. 2010 4 1;70(7):2644–2654. 20233876 10.1158/0008-5472.CAN-09-3644

[36] Thoma CR, Stroebel S, Rosch N, et al A high-throughput-compatible 3D microtissue co-culture system for phenotypic RNAi screening applications. J Biomol Screen. 2013 12;18(10):1330–1337. 24080258 10.1177/1087057113499071

[37] Thoma CR, Zimmermann M, Agarkova I, et al 3D cell culture systems modeling tumor growth determinants in cancer target discovery. Adv Drug Deliv Rev. 2014;69–70:29–41. 10.1016/j.addr.2014.03.001 24636868

[38] Ahn SM, Simpson RJ Body fluid proteomics: prospects for biomarker discovery. Proteomics Clin Appl. 2007 9;1(9):1004–1015. 21136753 10.1002/prca.200700217

[39] Haslene-Hox H, Madani A, Berg KC, et al Quantification of the concentration gradient of biomarkers between ovarian carcinoma interstitial fluid and blood. BBA Clin. 2014;2:18–23. 26673827 10.1016/j.bbacli.2014.08.002 PMC4633919

[40] Karsdal MA, Nielsen MJ, Sand JM, et al Extracellular matrix remodeling: the common denominator in connective tissue diseases. Possibilities for evaluation and current understanding of the matrix as more than a passive architecture, but a key player in tissue failure. Assay Drug Dev Technol. 2013 3;11(2):70–92. 23046407 10.1089/adt.2012.474 PMC3593693

[41] Kraning-Rush CM, Reinhart-King CA Controlling matrix stiffness and topography for the study of tumor cell migration. Cell Adh Migr. 2012 May–Jun;6(3):274–279. 22863740 10.4161/cam.21076 PMC3427241

[42] Wolf K, Friedl P Extracellular matrix determinants of proteolytic and non-proteolytic cell migration. Trends Cell Biol. 2011 12;21(12):736–744. 22036198 10.1016/j.tcb.2011.09.006

[43] Flach EH, Rebecca VW, Herlyn M, et al Fibroblasts contribute to melanoma tumor growth and drug resistance. Mol Pharm. 2011 12 5;8(6):2039–2049. 22067046 10.1021/mp200421k PMC3235959

[44] Imamura Y, Mukohara T, Shimono Y, et al Comparison of 2D- and 3D-culture models as drug-testing platforms in breast cancer. Oncol Rep. 2015 4;33(4):1837–1843. 25634491 10.3892/or.2015.3767

[45] Patra B, Peng CC, Liao WH, et al Drug testing and flow cytometry analysis on a large number of uniform sized tumor spheroids using a microfluidic device. Sci Rep. 2016 2;15(6):21061. 10.1038/srep21061 PMC4753452 26877244

[46] Hoffmann OI, Ilmberger C, Magosch S, et al Impact of the spheroid model complexity on drug response. J Biotechnol. 2015 7 10;205:14–23. 25746901 10.1016/j.jbiotec.2015.02.029

[47] Theodoraki MA, Rezende CO Jr., Chantarasriwong O, et al Spontaneously-forming spheroids as an in vitro cancer cell model for anticancer drug screening. Oncotarget. 2015 8 28;6(25):21255–21267. 26101913 10.18632/oncotarget.4013 PMC4673263

[48] Hwang H, Kim EK, Park J, et al RhoA and Rac1 play independent roles in lysophosphatidic acid-induced ovarian cancer chemotaxis. Integr Biol (Camb). 2014 3;6(3):267–276. 24469268 10.1039/c3ib40183a

[49] Olivo C, Vanni C, Mancini P, et al Distinct involvement of cdc42 and RhoA GTPases in actin organization and cell shape in untransformed and Dbl oncogene transformed NIH3T3 cells. Oncogene. 2000 3 9;19(11):1428–1436. 10723134 10.1038/sj.onc.1203440

[50] Su S, Li Y, Luo Y, et al Proteinase-activated receptor 2 expression in breast cancer and its role in breast cancer cell migration. Oncogene. 2009 8 27;28(34):3047–3057. 19543320 10.1038/onc.2009.163 PMC2733915

[51] Kim EK, Park JM, Lim S, et al Activation of AMP-activated protein kinase is essential for lysophosphatidic acid-induced cell migration in ovarian cancer cells. J Biol Chem. 2011 7 8;286(27):24036–24045. 21602274 10.1074/jbc.M110.209908 PMC3129185

[52] Tkach V, Bock E, Berezin V The role of RhoA in the regulation of cell morphology and motility. Cell Motil Cytoskeleton. 2005 5;61(1):21–33. 15776463 10.1002/cm.20062

[53] Guo F, Zheng Y Involvement of Rho family GTPases in p19Arf- and p53-mediated proliferation of primary mouse embryonic fibroblasts. Mol Cell Biol. 2004 2;24(3):1426–1438. 14729984 10.1128/MCB.24.3.1426-1438.2004 PMC321455

[54] Hirakawa M, Oike M, Karashima Y, et al Sequential activation of RhoA and FAK/paxillin leads to ATP release and actin reorganization in human endothelium. J Physiol London. 2004 7 15;558(2):479–488. 15155793 10.1113/jphysiol.2004.065334 PMC1664968

[55] Appert-Collin A, Bennasroune A, Jeannesson P, et al Role of LRP-1 in cancer cell migration in 3-dimensional collagen matrix. Cell Adh Migr. 2017 7 4;11(4):316–326. 27463962 10.1080/19336918.2016.1215788 PMC5569966

